# Supplementary material for: The G3BP stress-granule proteins reinforce the integrated stress response translation programme
Source: Nat Cell Biol. 2025 Dec 19;28(1):135–48. doi: 10.1038/s41556-025-01834-3 (PMC12807861; doi:10.1038/s41556-025-01834-3)
Supplement: Supplementary file 1 — Reporting Summary [file 41556_2025_1834_MOESM1_ESM.pdf]

nature portfolio

Corresponding author(s): David Bartel

Last updated by author(s): Sep 3, 2025

Reporting Summary

Nature Portfolio wishes to improve the reproducibility of the work that we publish. This form provides structure for consistency and transparency in reporting. For further information on Nature Portfolio policies, see our Editorial Policies and the Editorial Policy Checklist.

Statistics

For all statistical analyses, confirm that the following items are present in the figure legend, table legend, main text, or Methods section.

n/a	Confirmed	
☐	☒	The exact sample size (*n*) for each experimental group/condition, given as a discrete number and unit of measurement
☐	☒	A statement on whether measurements were taken from distinct samples or whether the same sample was measured repeatedly
☐	☒	The statistical test(s) used AND whether they are one- or two-sided<br>*Only common tests should be described solely by name; describe more complex techniques in the Methods section.*
☒	☐	A description of all covariates tested
☐	☒	A description of any assumptions or corrections, such as tests of normality and adjustment for multiple comparisons
☐	☒	A full description of the statistical parameters including central tendency (e.g. means) or other basic estimates (e.g. regression coefficient) AND variation (e.g. standard deviation) or associated estimates of uncertainty (e.g. confidence intervals)
☐	☒	For null hypothesis testing, the test statistic (e.g. F, t, r) with confidence intervals, effect sizes, degrees of freedom and P value noted<br>*Give P values as exact values whenever suitable.*
☒	☐	For Bayesian analysis, information on the choice of priors and Markov chain Monte Carlo settings
☒	☐	For hierarchical and complex designs, identification of the appropriate level for tests and full reporting of outcomes
☐	☒	Estimates of effect sizes (e.g. Cohen's d, Pearson's r), indicating how they were calculated

Our web collection on statistics for biologists contains articles on many of the points above.

Software and code

Policy information about availability of computer code

Data collection	Microscopy images were acquired using Andor spinning disk confocal running iQ acquisition software and Metamorph acquisition software , Nikon Ti automated inverted microscope running MetaMorph acquisition software, and a Zeiss LSM 980 with Airyscan 2 Laser Scanning Confocal imaging system running Zen Blue software. Western Blots were imaged using Odyssey CLx imaging system by LI-COR running Image Studio software. Luciferase readings were acquired using GloMax microplate reader running Glomax software. RT-qPCR measurements were made using QuantStudio 6 Pro RT-PCR System running QuantStudio software.
Data analysis	Images were analyzed using IMARIS software. Westerns were analyzed using FIJI/ImageJ version 2.9.0/1.53u. Statistical analyses were performed using RStudio version 2023.09.0+463. Some simpler statistical analyses were performed using Microsoft Excel version 16.78.3.

For manuscripts utilizing custom algorithms or software that are central to the research but not yet described in published literature, software must be made available to editors and reviewers. We strongly encourage code deposition in a community repository (e.g. GitHub). See the Nature Portfolio guidelines for submitting code & software for further information.

Data

Policy information about availability of data

All manuscripts must include a data availability statement. This statement should provide the following information, where applicable:

- Accession codes, unique identifiers, or web links for publicly available datasets
- A description of any restrictions on data availability
- For clinical datasets or third party data, please ensure that the statement adheres to our policy

Raw sequencing data for all ribo-seq and SG purification experiments in this publication are available for download at GEO: GSE256237

Research involving human participants, their data, or biological material

Policy information about studies with human participants or human data. See also policy information about sex, gender (identity/presentation), and sexual orientation and race, ethnicity and racism.

Reporting on sex and gender	No human participants, their data, or their biological material were used for this study
Reporting on race, ethnicity, or other socially relevant groupings	No human participants, their data, or their biological material were used for this study
Population characteristics	No human participants, their data, or their biological material were used for this study
Recruitment	No human participants, their data, or their biological material were used for this study
Ethics oversight	No human participants, their data, or their biological material were used for this study

Note that full information on the approval of the study protocol must also be provided in the manuscript.

Field-specific reporting

Please select the one below that is the best fit for your research. If you are not sure, read the appropriate sections before making your selection.

☒ Life sciences ☐ Behavioural & social sciences ☐ Ecological, evolutionary & environmental sciences

For a reference copy of the document with all sections, see nature.com/documents/nr-reporting-summary-flat.pdf

Life sciences study design

All studies must disclose on these points even when the disclosure is negative.

Sample size	No methods were used to predetermine sample sizes. Sample sizes were determined based on expected variation, availability of resources, and what would be required to confidently determine presence or absence of statistical significance.
Data exclusions	No data were excluded from analyses
Replication	All data were replicated with a number of replicates indicated in each figure legend and in data points shown in plots, with additional information on replicates shown in methods.
Randomization	No randomization was performed for this study. It was not necessary as quantifications were performed in an unbiased manner, with identical thresholds across all compared samples.
Blinding	Blinding was not relevant to the study as all quantifications/assessments were done using a consistent controlled set of criteria across compared samples.

Reporting for specific materials, systems and methods

We require information from authors about some types of materials, experimental systems and methods used in many studies. Here, indicate whether each material, system or method listed is relevant to your study. If you are not sure if a list item applies to your research, read the appropriate section before selecting a response.

Materials & experimental systems

n/a	Involved in the study	
☐	☒	Antibodies
☐	☒	Eukaryotic cell lines
☒	☐	Palaeontology and archaeology
☒	☐	Animals and other organisms
☒	☐	Clinical data
☒	☐	Dual use research of concern
☒	☐	Plants

Methods

n/a	Involved in the study	
☒	☐	ChIP-seq
☒	☐	Flow cytometry
☒	☐	MRI-based neuroimaging

Antibodies

Antibodies used

Antibodies used were: anti-CAPRIN (151121-1-AP), anti-G3BP1 (AB_398438), anti-G3BP2 (A302-040A), anti-GAPDH (60004-1-Ig), anti-PABP1 (CST - 4992), anti-eIF4G (CST – 2498), Goat anti-Rabbit secondary IRDye 800 (926-32211), Donkey anti-Mouse IRDye 680 (926-68072), Goat anti-Rabbit Cy5 (Invitrogen - A10523)

Validation

G3BP1, G3BP2, and CAPRIN antibodies were validated using AID depletion in this study and all antibodies used were validated by their supplies.
CAPRIN1 - https://www.ptglab.com/products/CAPRIN1-Antibody-15112-1-AP.htm
G3BP1 - https://www.bdbiosciences.com/en-us/products/reagents/microscopy-imaging-reagents/immunofluorescence-reagents/purified-mouse-anti-human-g3bp.611126
G3BP2 - https://www.fortislife.com/products/primary-antibodies/rabbit-anti-g3bp2-antibody/BETHYL-A302-040
GAPDH - https://www.ptglab.com/products/GAPDH-Antibody-60004-1-Ig.htm
PABP1 - https://www.cellsignal.com/products/primary-antibodies/pabp1-antibody/4992#application-type-if
eIF4G - https://www.cellsignal.com/products/primary-antibodies/eif4g-antibody/2498
Goat anti-Rabbit secondary IRDye 800 - https://www.licor.com/bio/reagents/irdye-800cw-goat-anti-rabbit-igg-secondary-antibody
Donkey anti-Mouse IRDye 680 - https://www.licor.com/bio/reagents/irdye-680rd-donkey-anti-mouse-igg-secondary-antibody
Goat anti-Rabbit Cy5 - https://www.thermofisher.com/antibody/product/Goat-anti-Rabbit-IgG-H-L-Cross-Adsorbed-Secondary-Antibody-Polyclonal/A10523

Eukaryotic cell lines

Policy information about cell lines and Sex and Gender in Research

Cell line source(s)

HCT116 cells were obtained from the Richard Young Lab
U2OS cells were obtained from ATCC
All derived cell lines used in this study were created for this study

Authentication

Cell line genotypes were confirmed by PCR and western for modified gene products

Mycoplasma contamination

Cell lines tested negative for mycoplasma

Commonly misidentified lines
(See ICLAC register)

None of these lines were used for this study

Plants

Seed stocks

No plants were used for this study

Novel plant genotypes

No plants were used for this study

Authentication

No plants were used for this study
