# Supplementary figures and images for: The G3BP stress-granule proteins reinforce the integrated stress response translation programme
Source: Nat Cell Biol. 2025 Dec 19;28(1):135–48. doi: 10.1038/s41556-025-01834-3 (PMC12807861; doi:10.1038/s41556-025-01834-3)

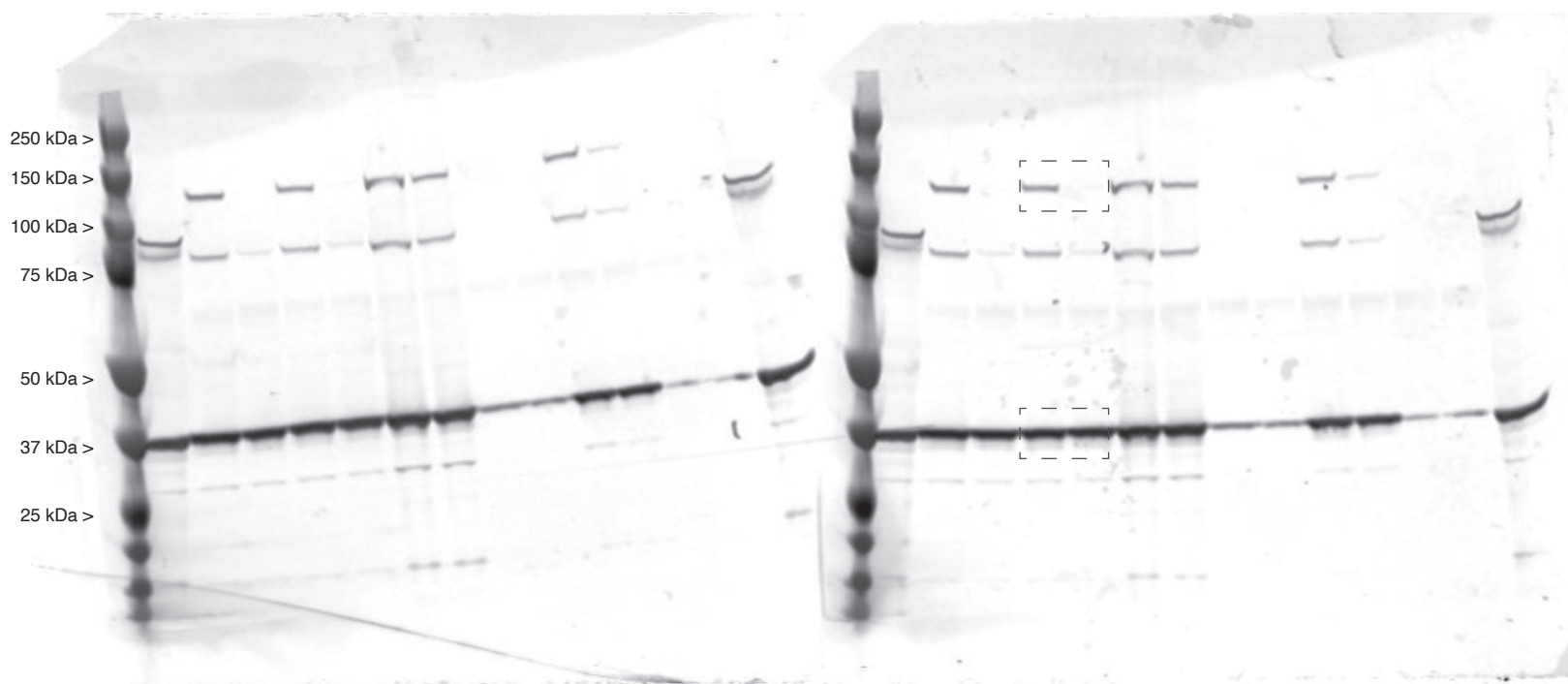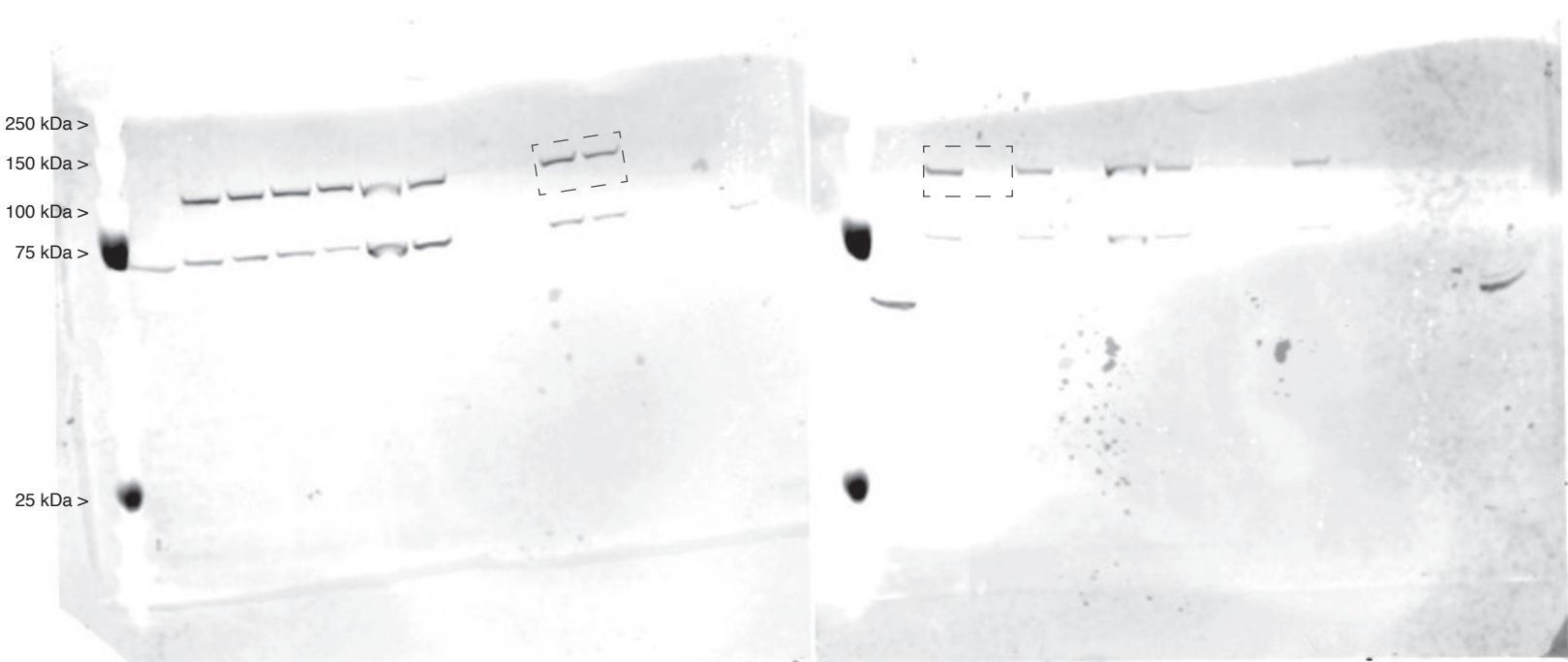

Uncropped images from Fig 2C and 2E

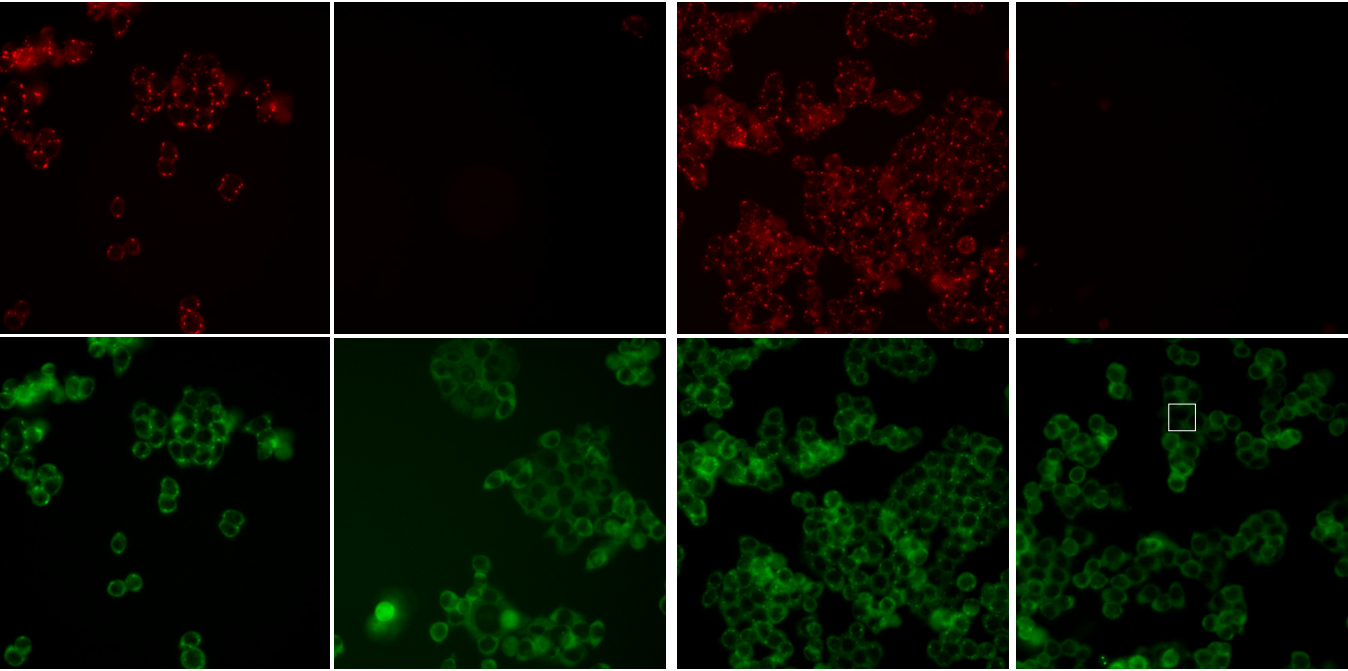

Supplement: Supplementary file 7 — Unprocessed western blots and images. [file 41556_2025_1834_MOESM7_ESM.pdf]

Uncropped images from Fig 4B

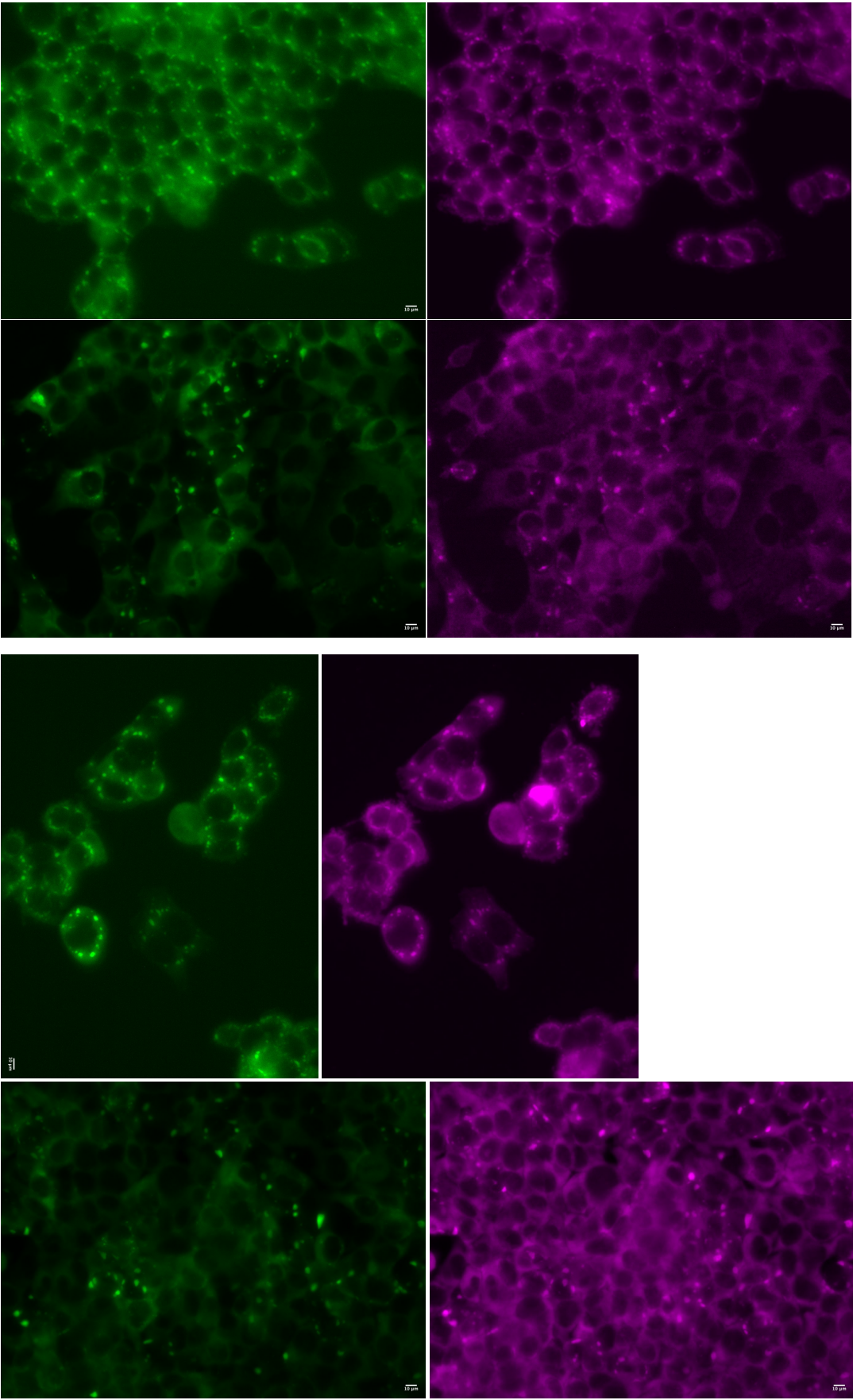

Supplement: Supplementary file 8 — Unprocessed western blots and images. [file 41556_2025_1834_MOESM8_ESM.pdf]

Uncropped images from Fig 5A

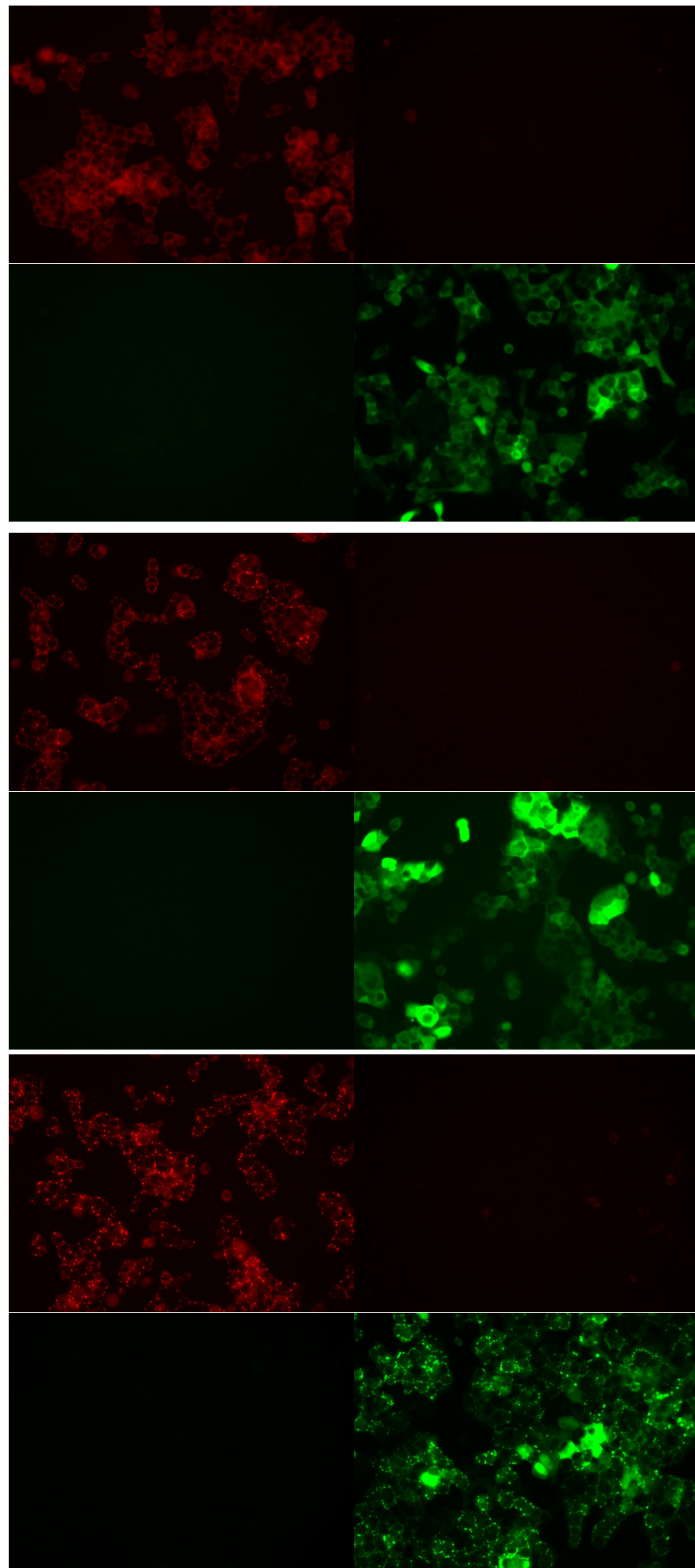

Supplement: Supplementary file 9 — Unprocessed western blots and images. [file 41556_2025_1834_MOESM9_ESM.pdf]

Uncropped images from Fig 6B

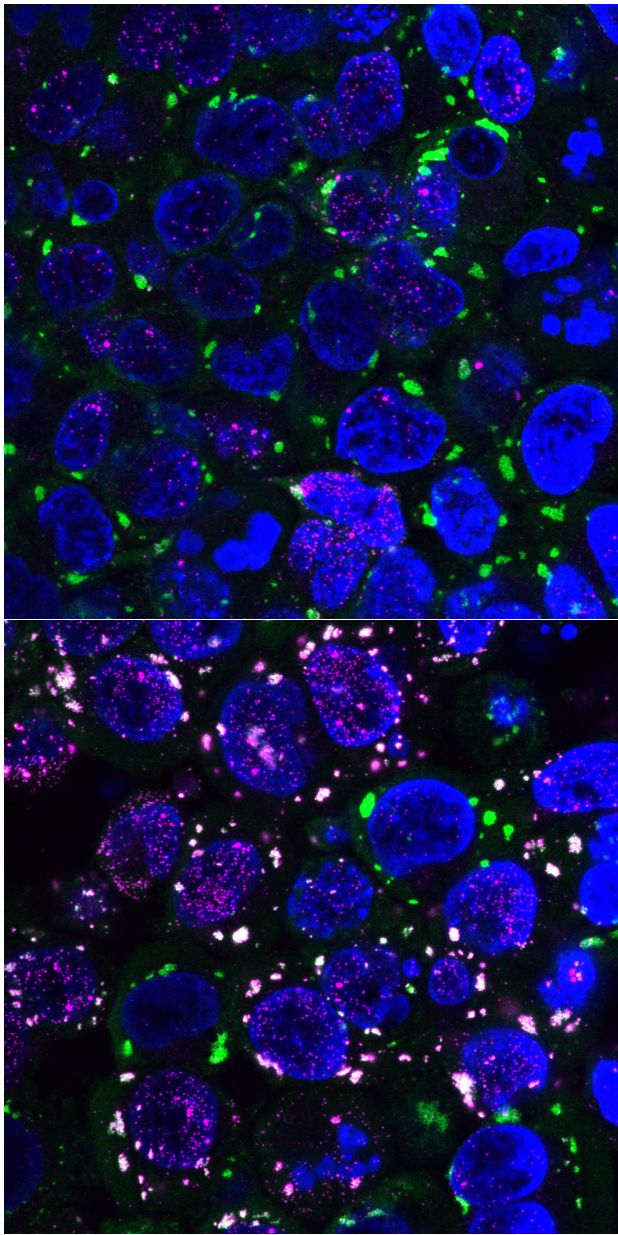

Supplement: Supplementary file 10 — Unprocessed western blots and images. [file 41556_2025_1834_MOESM10_ESM.pdf]

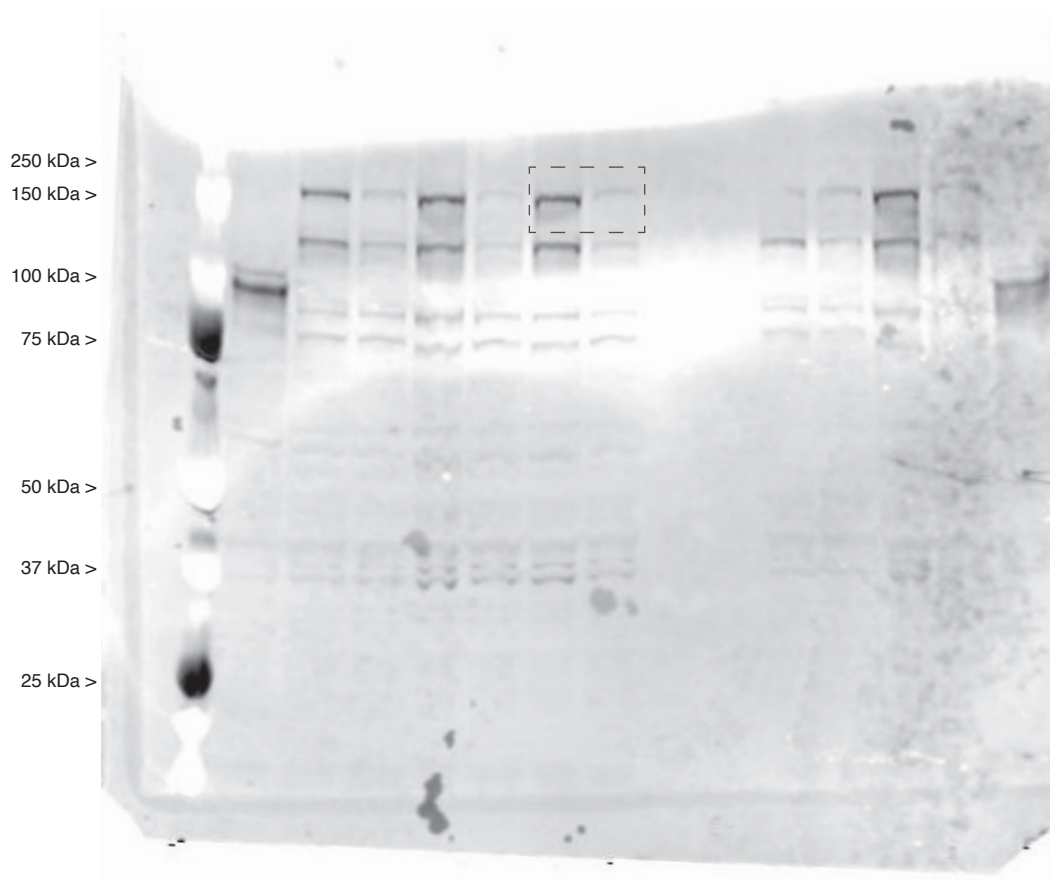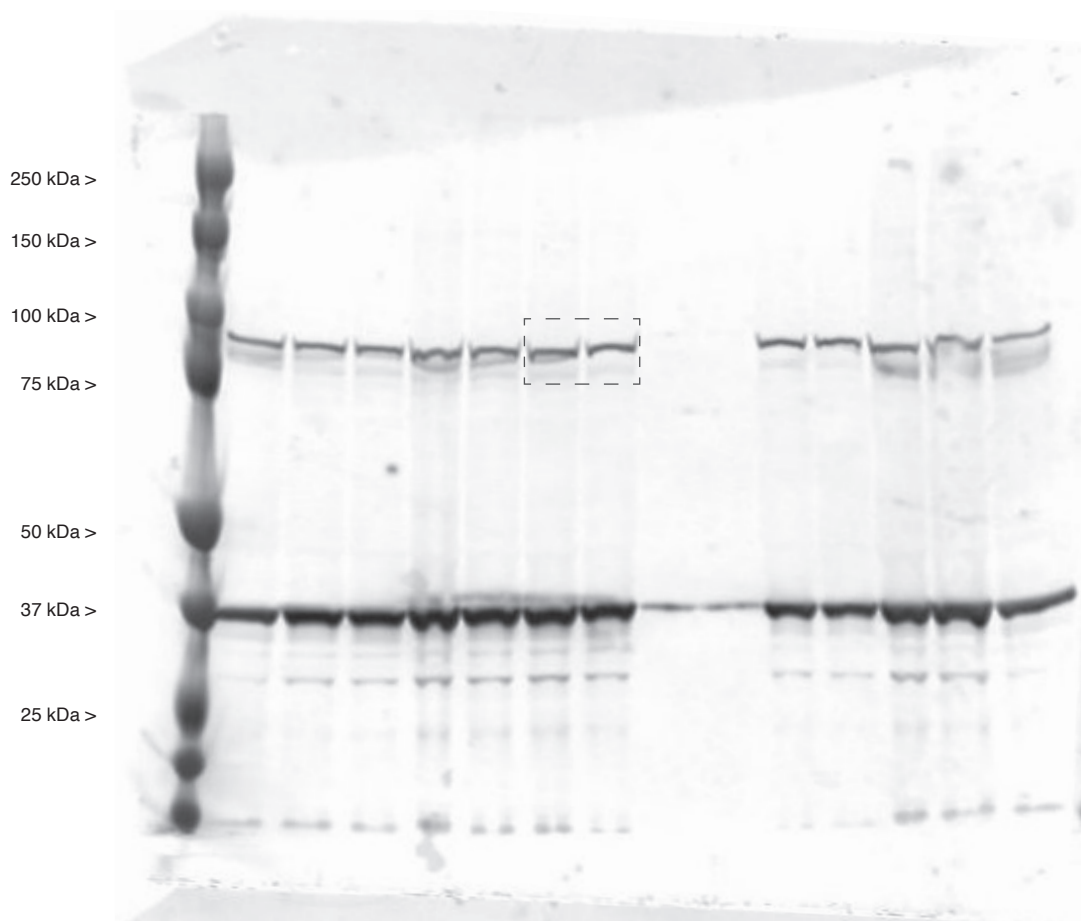

Supplement: Supplementary file 11 — Unprocessed western blots and images. [file 41556_2025_1834_MOESM11_ESM.pdf]

Uncropped blots from Extended Data Fig 6A and 6C

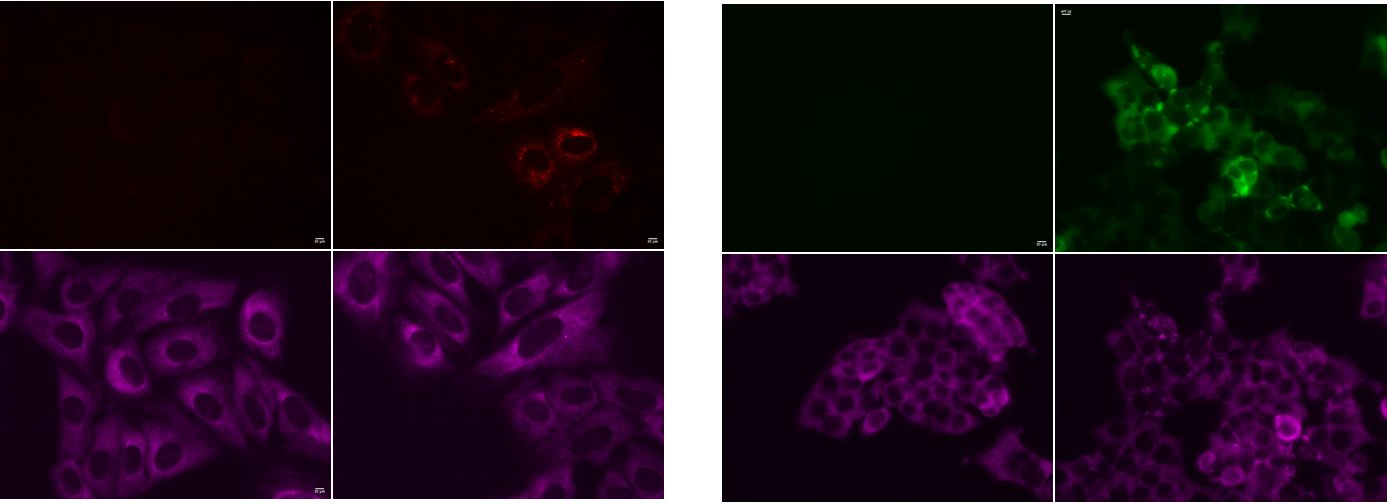

Supplement: Supplementary file 12 — Unprocessed western blots and images. [file 41556_2025_1834_MOESM12_ESM.pdf]

Uncropped blots from Extended Data Fig 8A and 8C

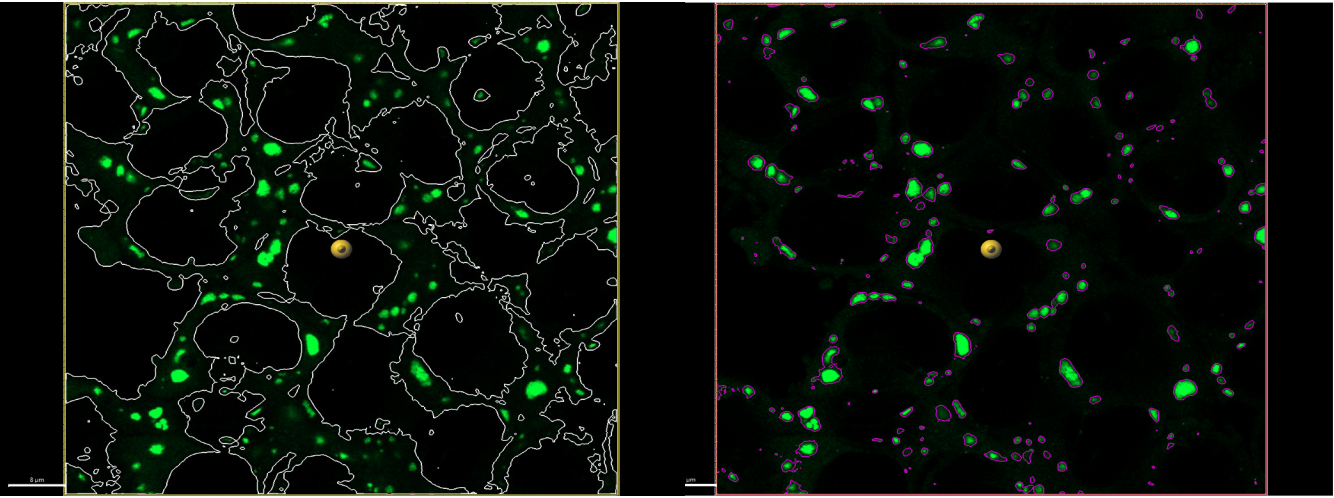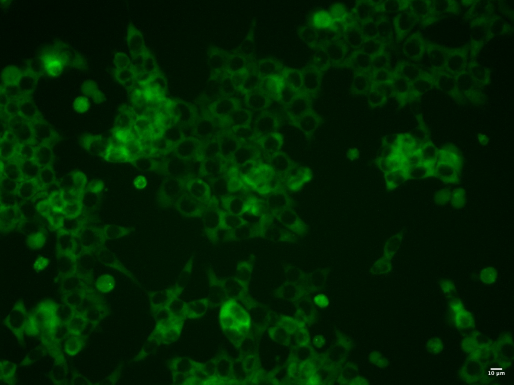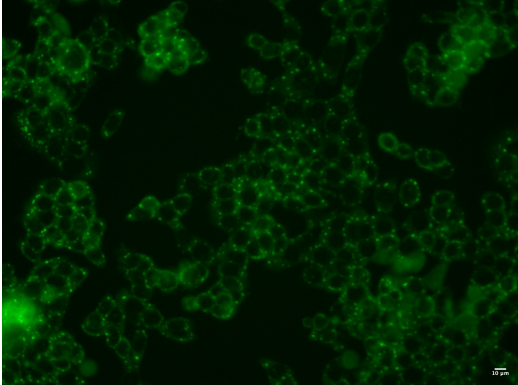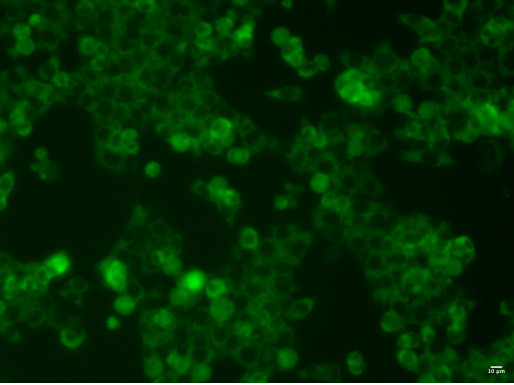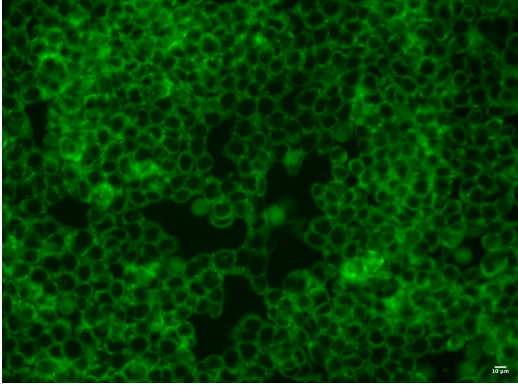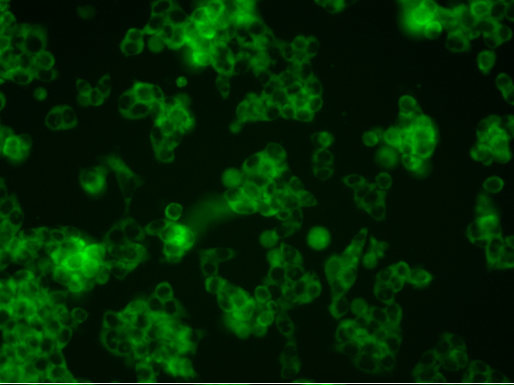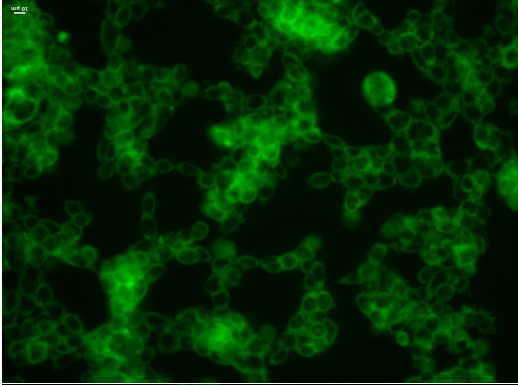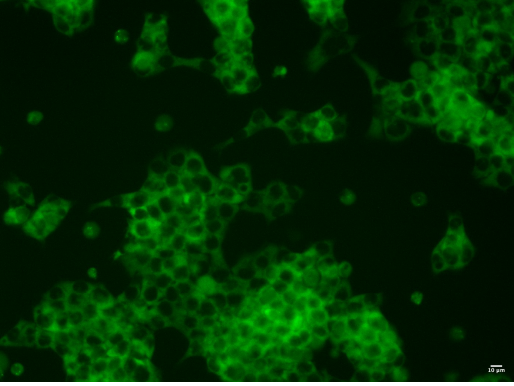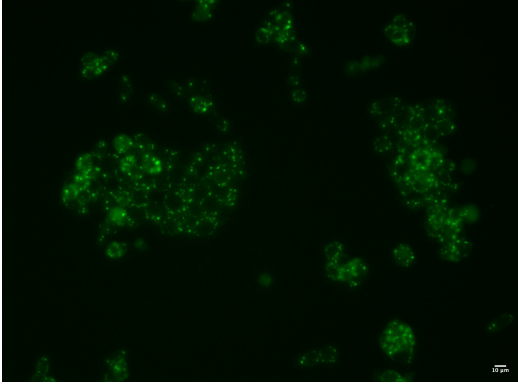

Supplement: Supplementary file 13 — Unprocessed western blots and images. [file 41556_2025_1834_MOESM13_ESM.pdf]

Uncropped blots from Extended Data Fig 9A and 9C

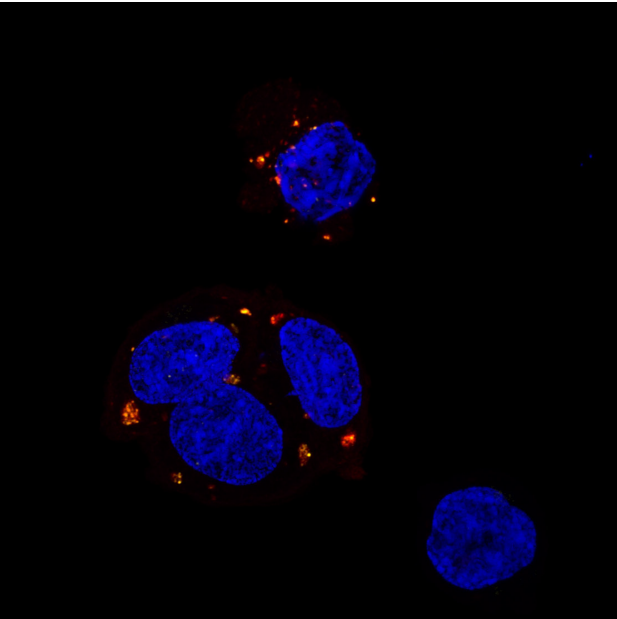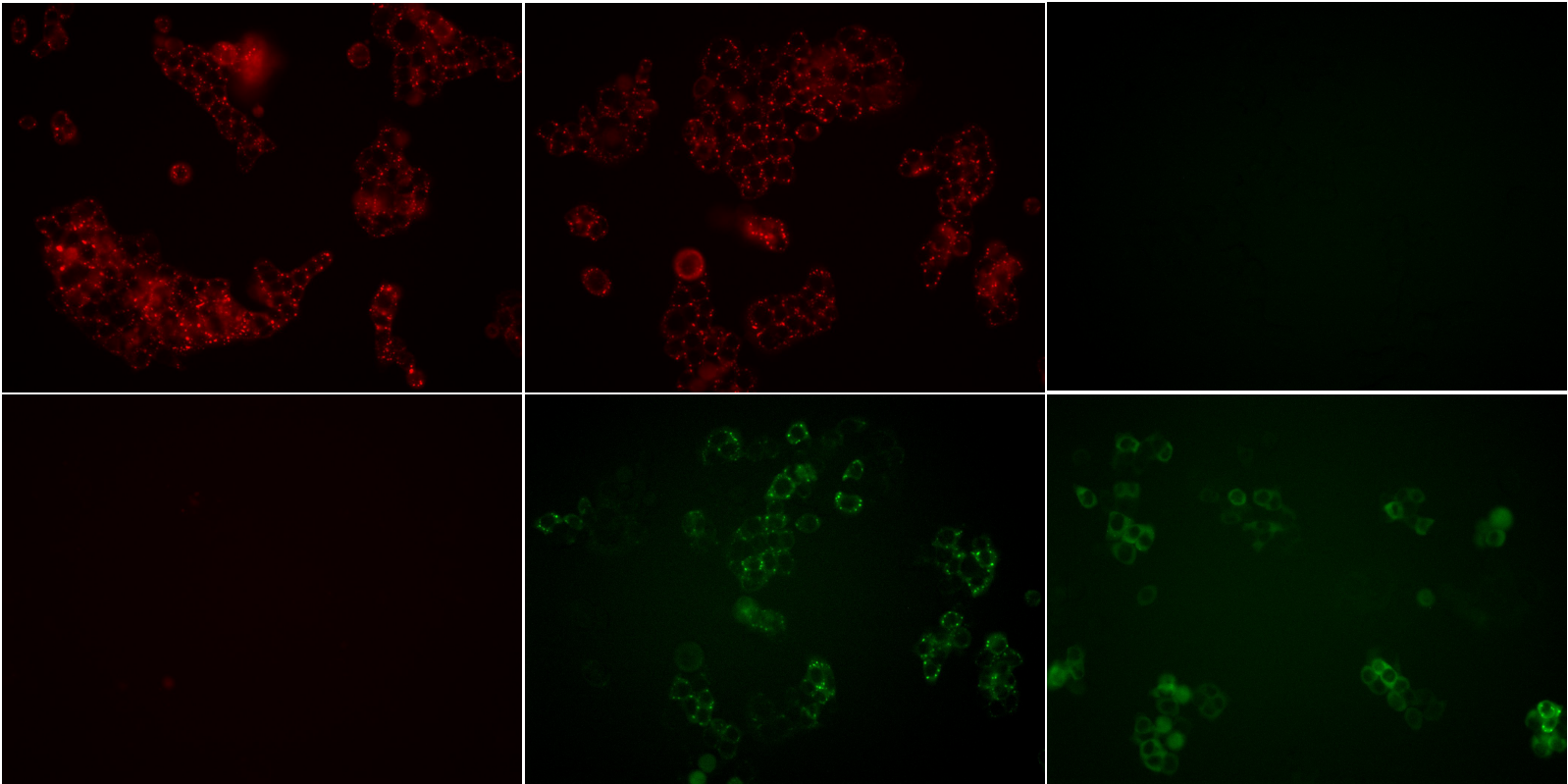

Supplement: Supplementary file 14 — Unprocessed western blots and images. [file 41556_2025_1834_MOESM14_ESM.pdf]
